# Human–nature connectedness as a pathway to sustainability: A global meta‐analysis

**DOI:** 10.1111/conl.12852

**Published:** 2021-11-21

**Authors:** Gladys Barragan‐Jason, Claire de Mazancourt, Camille Parmesan, Michael C. Singer, Michel Loreau

**Affiliations:** ^1^ Theoretical and Experimental Ecology Station CNRS Moulis France; ^2^ Biological and Marine Sciences University of Plymouth Plymouth UK; ^3^ Department of Geological Sciences University of Texas at Austin Austin Texas

**Keywords:** environmental education, human health, human–nature connectedness, meta‐analysis, nature conservation, nature‐based solutions, people and nature, proenvironmental behavior, sustainability

## Abstract

Internationally agreed sustainability goals are being missed. Here, we conduct global meta‐analyses to assess how the extent to which humans see themselves as part of nature—known as *human–nature connectedness* (HNC)—can be used as a leverage point to reach sustainability. A meta‐analysis of 147 correlational studies shows that individuals with high HNC had more pronature behaviours and were significantly healthier than those with low HNC. A meta‐analysis of 59 experimental studies shows significant increases in HNC after manipulations involving contact with nature and mindfulness practices. Surprisingly, this same meta‐analysis finds no significant effect of environmental education on HNC. Thus, HNC is positively linked to mind‐sets that value sustainability and behaviours that enhance it. Further, we argue that HNC can be enhanced by targeted practices, and we identify those most likely to succeed. Our results suggest that enhancing HNC, via promotion of targeted practices, can improve sustainability and should be integrated into conservation policy.

## 1 INTRODUCTION

Anthropogenic climate change and biodiversity loss are major threats not only to nonhuman living beings but also to our own survival (Brondizio et al., 2019; Díaz et al., 2019; Pachauri & Meyer, 2014; Shukla et al., 2019). There appears to be international consensus to better preserve nature (e.g., the Convention on Biological Diversity; CBD, 2011), to limit global warming (e.g., the Paris Agreements) and to create a sustainable, equitable world (e.g., the Sustainable Development Goals). Despite this appearance, actions on all three fronts remain limited and sustainability targets have so far failed to be achieved (Adenle, 2012; Buchanan et al., 2020). Why do citizens and governments still find it hard to consider that the health and well‐being of humans depends upon the health of the natural world (Brondizio et al., 2019; Pachauri & Meyer, 2014; Shukla et al., 2019)?

**FIGURE 1:**
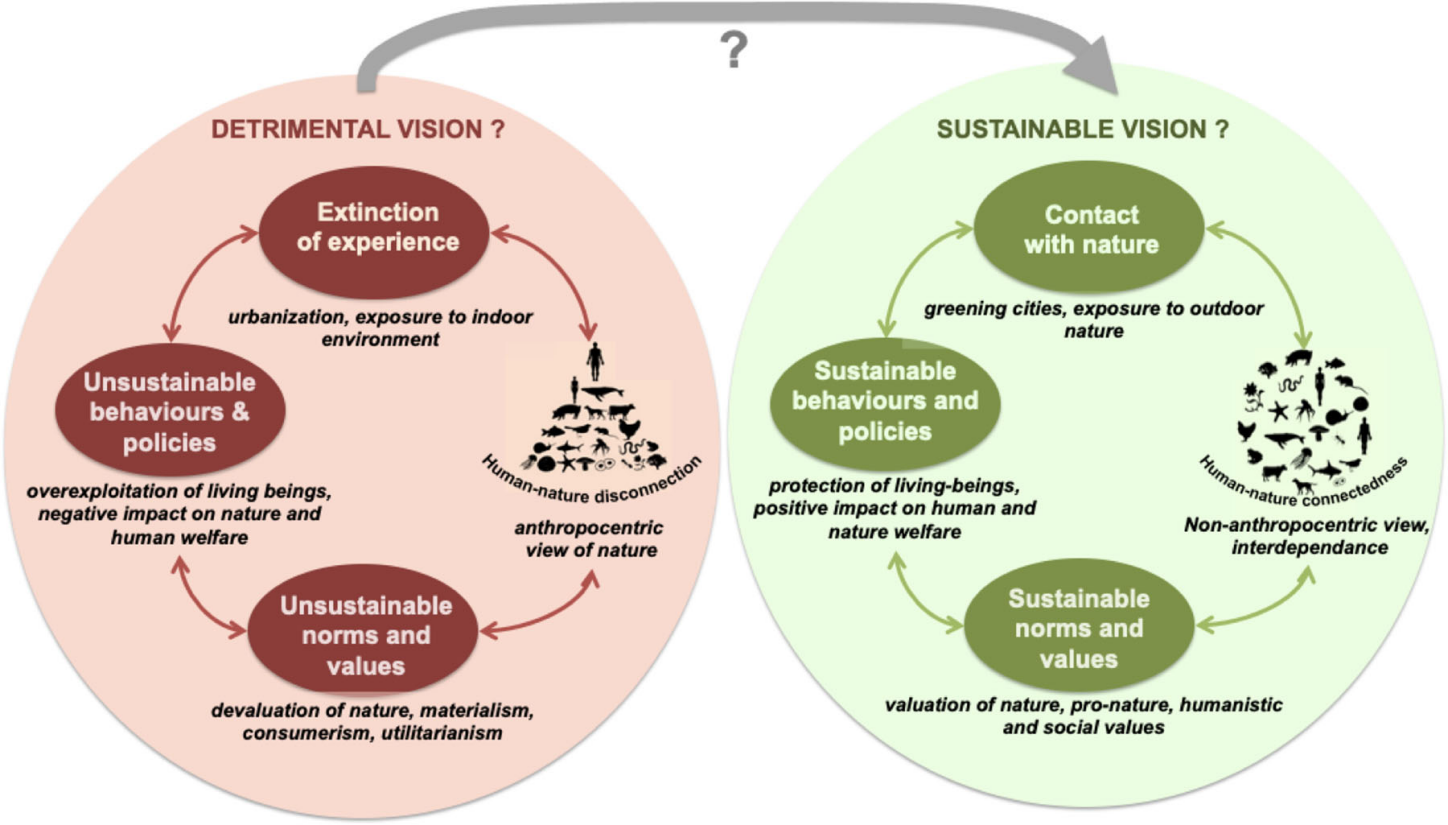
Hypothetical representation of a vicious circle generated by a detrimental worldview (left) and a virtuous circle generated by a sustainable worldview (right). We assess whether and how human–nature connectedness can be used to switch from a detrimental to a sustainable worldview at both individual and societal levels

One possible explanation is cultural (Mayer, 2018). Humans in many societies perceive a clear discontinuity between the inner worlds of themselves and those of other living beings. This view, explicitly advocated by influential modern philosophers such as Kant (1784), has led people to consider themselves disconnected from, and dominant over, the rest of nature. Another explanation is urbanization of the world's human population. Today, more than 55% of people live in urban areas (https://www.un.org/en/sections/issues‐depth/population/index.html), a major consequence of which is disconnection of people from experience of natural habitats (Miller, 2005). The combination of psychological and physical disconnects from the natural world can result in devaluation of nature (Mayer, 2018), thereby legitimizing and facilitating destructive practices toward nature by individuals and societies (Figure 1).

What can be done to modify these destructive trends? International scientific assessments, such as the MA, IPCC, and IPBES, indicate that healthy natural systems are crucial to reach sustainability (Brondizio et al., 2019; Shukla et al., 2019; Watson et al., 2005). The IPBES further claims that sustainable goals will not be achieved without a “transformative change” including an increase in “awareness of connectivity in the environmental crisis and new norms regarding interactions between humans and nature” (Figure 1). A key societal trait that is relevant to achieving sustainability (Riechers et al., 2021) is Human–Nature Connectedness (HNC). Leopold (1949) gave one of the first definitions of HNC when writing: “We abuse land because we regard it as a commodity belonging to us. When we see land as a community to which we belong, we may begin to use it with love and respect” (see Appendix 1). HNC has been described (Schultz, 2002), defined and conceptualized in different, often complex, ways (Supplementary information [SI] Appendices 1 and 2). Despite these differences, robust and validated HNC metrics have been shown to be highly correlated with one another (Balundė et al., 2019; Brügger et al., 2011; Mayer & Frantz, 2004; Nisbet et al., 2009; Perrin & Benassi, 2009; Tam, 2013), which suggests that they are different expressions of a common underlying construct (Capaldi et al., 2014; Tam, 2013). Here we define HNC as the extent to which humans see themselves as part of nature, and we use it as an umbrella term for the different concepts and metrics that address this relatively simple and broad definition.

A number of recent studies found that estimates of HNC were positively correlated both with proenvironmental behaviour (Mackay & Schmitt, 2019; Vesely et al., 2021; Whitburn et al., 2020) and with human welfare (Capaldi et al., 2014; Pritchard et al., 2020). Due to paucity of data, however, prior syntheses of the roles of HNC were forced to lump studies with disparate methodologies, whether they be experimental or correlational, and whether or not they used metrics of HNC that had been validated. Here, we take advantage of the rapidly increasing literature to restrict our database to studies using at least one of the validated quantitative metrics of HNC (Clayton, 2003; Dunlap et al., 2000; Mayer & Frantz, 2004; Nisbet et al., 2009; Schultz, 2002) and to distinguish between experimental and correlational studies. Thus, for the first time, we provide a robust, coherent, and global synthesis of the HNC literature with separate meta‐analyses of experimental and correlational studies (Figure S1 and Table S1). This allows us to identify practices that have the potential for increasing HNC, and investigate whether HNC is a key element of sustainability, in particular nature conservation and human welfare.

## 2 METHODS

### 2.1 Data search and inclusion

We followed the PRISMA guidelines to conduct a systematic review of the literature (O'Dea et al., 2021). We searched previous papers assessing HNC on Web of Science (WoS) and PubMed from 1900 to 2020 with search terms corresponding to the various HNC metrics used in previous published reviews, opinions and meta‐analyses (see details in Table S1), and obtained 2098 citations (see Figure S1 for PRISMA flow diagram).

Only *empirical, quantitative and peer‐reviewed published* papers that were *written in English* were made eligible for inclusion. Researchers have developed several hundred constructs and metrics of HNC that tackle one or several aspects of HNC (e.g., cognitive and affective components). Despite differences in how HNC is conceptualized, five metrics have been shown to robustly assess HNC and to be highly correlated with one another (Balundė et al., 2019; Brügger et al., 2011; Mayer & Frantz, 2004; Nisbet et al., 2009; Perrin & Benassi, 2009; Tam, 2013). We focused on these five metrics (for a detailed description of HNC scales, see SI Appendices 2 and 3): the New Environmental Paradigm (NEP; Dunlap et al., 2000), the Inclusion of Nature in the Self (INS; Schultz, 2002), the Environmental Identity Scale (EID; Clayton, 2003), the Connectedness to Nature Scale (CNS; Mayer & Frantz, 2004), and the Nature Relatedness scale (NR; Nisbet et al., 2009). While NEP, INS, and CNS attempt to measure the cognitive dimension of HNC, NR, and EID include both cognitive and affective components (Clayton, 2003; Nisbet et al., 2009). Papers measuring *at least one widely used and repeatable HNC* measure (*NEP, INS, EID, CNS, or NR)* and *at least one factor* (correlational and/or experimental studies) were included.

At the end of the screening, 124 publications met the eligibility criteria. For all studies, we extracted the *number of participants*, *region* (Africa, Asia, Europe, North America, South America, and Oceania), *country*, *gender ratio* (**>**60% female, between 40 and 60% female, <40 % female) and *age group* (<18 years, between 18 and 25 years, between 26 and 40 years, >40 years) as well as duration of the intervention (≤1 day, ≥2 days) and time of posttest (immediately after intervention, ≥2 weeks after intervention) when appropriate. For correlational studies (107 papers), we extracted Pearson's correlation coefficients between HNC and the factors of interest. For experimental studies (35 papers), we extracted HNC means and standard deviations for treatment and control groups. When correlations, means, or SDs were missing from the paper, we emailed the corresponding author. Overall, we obtained a total of 1080 effects sizes from 198 studies (i.e., sample) from 124 papers involving 69,763 participants to perform the meta‐analyses.

### 2.2 Statistical analysis

All statistical analyses were performed in R (4.0). For experimental data, we performed the meta‐analysis on Standardized Means Difference (SMD; Hedges’ g). For correlational data, meta‐analyses were done on Pearson's correlations after Fisher transformations. All estimates were calculated for each factor with the rma.mv function from the metaphor package (Viechtbauer & Viechtbauer, 2015) in R, which permits to fit meta‐analytic multivariate fixed‐ and random/mixed‐effects models with or without factors via linear (mixed‐effects) models.

We accounted for nonindependence of data by including random effects at the *article level* (multiple data from the same paper), at the *study level* (multiple data from the same participant) and at the *estimate level* (multiple estimates within a study within a paper). Then, we transformed mean effect sizes (SMD and Fisher's *Z*) into mean R effect sizes. *Z* tests and omnibus tests were performed when appropriate. We provide additional methods and results in SI (i.e., funnel and forest plots in Figure S5 and Appendices 4–6).

## 3 RESULTS

### 3.1 Experimental studies

Existing experimental studies are strongly biased toward adults rather than children and high‐income industrialized countries (Figure S2). Among them, we identified six types of experimental designs: (1) exposure to real nature: direct contact with nature, either outdoors or indoors; (2) exposure to virtual nature: videos or pictures of nature; (3) mindfulness: focusing one's attention on one's inner self and one's environment in the present moment; (4) environmental education: exposure to naturalist, scientific and ecological knowledge of the natural world; (5) combination of exposure to real nature + environmental education and, (6) combination of exposure to real nature + mindfulness.

We combined two types of comparison: (1) sequential comparison of trait measurements made on the same participants before and after experimental treatment and (2) simultaneous comparison of control and treatment groups (see Methods and SI Appendix 4).

**FIGURE 2:**
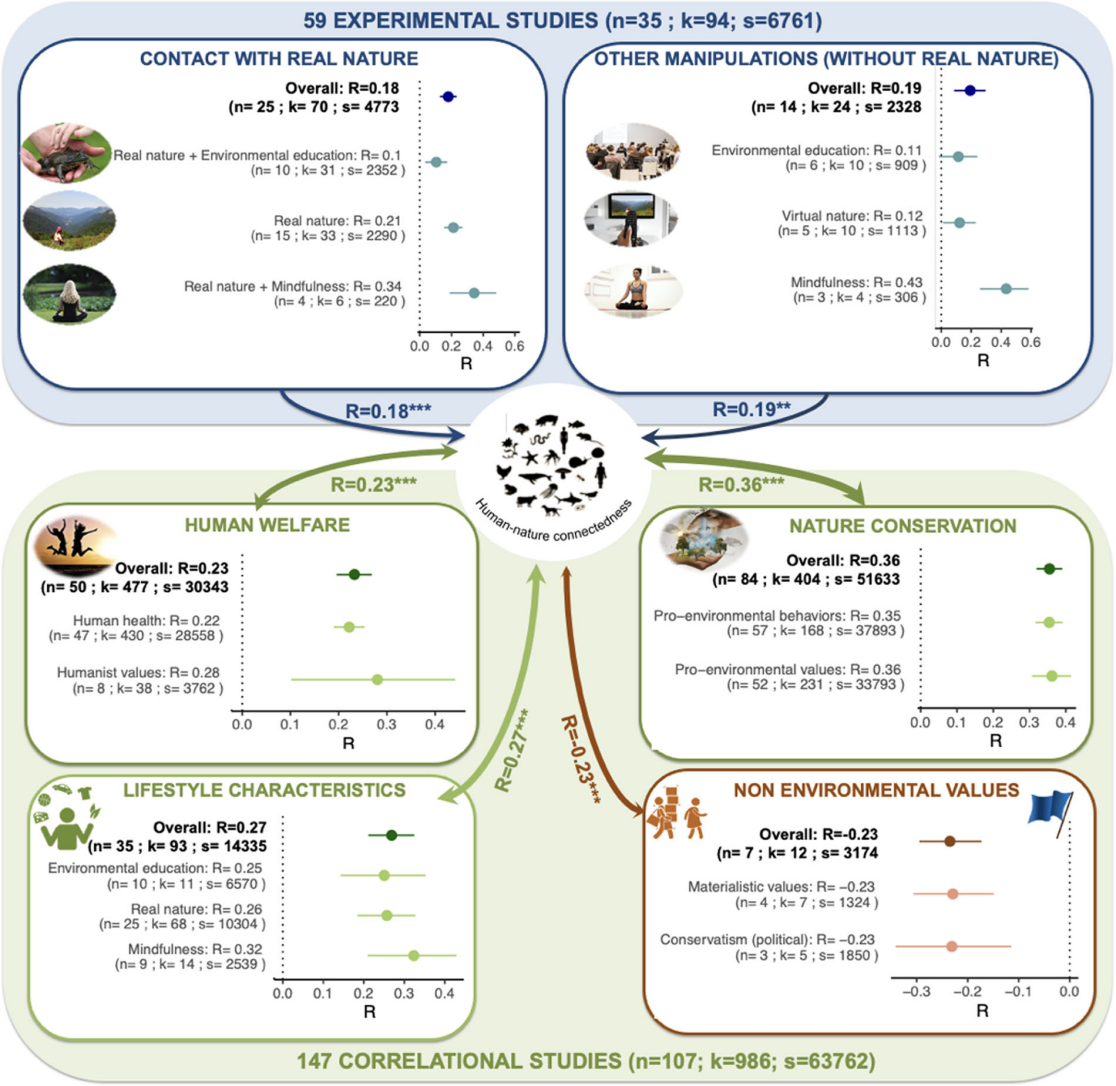
Results from meta‐analyses of experimental (blue background and text) and correlational (green and orange backgrounds and text) studies. Each dot represents the average R estimate for each factor obtained after transforming averaged standardized effect sizes (Fisher's *Z* for correlational data and Hedges’ g for experimental data) with *n*, *k*, and *s* referring to the number of papers, number of effect sizes and number of participants, respectively. Horizontal lines indicate the 95% confidence intervals for each factor and cross the vertical dot lines when nonsignificant (i.e., environmental education in experimental studies). Single‐headed arrows refer to causal relationships while double‐headed arrows refer to correlational links. Overall R estimates from each broad category are in bold. Details are provided in Methods, Appendix 5, Tables S2–S4, and Figure S4. Experimental studies show that exposure to nature and mindfulness practices improve HNC. Correlational studies confirmed experimental studies and show that HNC is positively linked to nature conservation and human welfare and negatively linked to nonenvironmental values. **p < 0.001; ***p < 0.0001

All experimental designs, except environmental education, affected HNC significantly and positively (Figure 2 and Table S2). The clearest positive effects on HNC were those of mindfulness, with or without experience of real nature, while the estimated positive effects of environmental education were low. Importantly, while both short (≤1 day) and long (≥2 days) interventions enhanced HNC in the short term (i.e., immediately after the intervention), long‐term effects (i.e., retention test after 2 weeks or more) were only observed after long interventions (Appendix 5 and Figure S3). Effect sizes did not differ significantly between HNC measures in experimental studies (Appendix 5, Figure S4, and Table S4).

Thus, experimental studies show that exposing individuals to nature, either real or virtual, does improve HNC. They also suggest exciting lines of future research to improve the efficiency of experimental programs designed to raise HNC by including mindfulness practices.

### 3.2 Correlational studies

Like experimental studies, correlational studies are strongly biased toward adults from industrialized countries. Our meta‐analysis shows that HNC is negatively correlated with materialism/consumerism and political conservatism, and positively correlated with the following suite of behaviours, opinions and personality traits: naturalist knowledge; time spent in natural outdoor spaces; engagement in mindfulness practices; proenvironmental values; humanistic values; happiness; and good health. In sum, individuals with high HNC had a deeper knowledge of nature, spent more time in natural outdoor spaces, engaged more in mindfulness practices and were happier and healthier than those with low HNC. It is not surprising, then, that individuals with high HNC displayed more proenvironmental values, expressed as concerns and attitudes toward their natural environment. They were also more humanistic, in the sense of more strongly expressing their moral responsibilities to other humans, and their sense of being part of communities within society (Figure 2; see Methods, SI Appendix 4 for detailed categories, Table S3 and Figures S4–S7).

Additionally, one‐third of the correlational papers (26 out of 108) addressed causal hypotheses using either structural equation models or path analysis. Although these approaches have limited ability to infer causality, these studies nonetheless suggest impacts of both contact with nature and mindfulness on HNC, in agreement with experimental studies (Figure 2 and Table S2). In the opposite direction of causality, they also suggest impacts of HNC on proenvironmental values, human well‐being, and health and proenvironmental behaviours.

Unlike experimental studies, effect sizes from correlational studies differed significantly between HNC metrics (Appendix 5 and Figure S4). Notably, HNC metrics that include both cognitive and affective aspects of HNC (i.e., EID, NR) showed significantly higher effect sizes than did HNC metrics with only cognitive components (i.e., CNS, NEP, INS; see Appendix 5, Figure S4, and Table S4).

Taken together, correlational studies support conclusions from experimental studies, but they further show that HNC is positively linked to values and behaviours that enhance ecological sustainability and well‐being, and negatively linked to nonenvironmental or antienvironmental values. Future experimental research contrasting the effects of interventions that do enhance or do not enhance HNC is needed to test causal relationships, particularly the hypothesis suggested by our meta‐analysis that raising HNC would enhance both human welfare and nature conservation.

## 4 DISCUSSION

Our meta‐analyses robustly show that the extent to which people feel part of nature can be enhanced by simple interventions involving contact with nature and mindfulness practices, at least in industrialized countries, which form the bulk of existing studies. They also show that validated HNC indices are positively linked to human welfare and nature conservation. Thus, improving HNC through contact with nature and mindfulness can be a valuable way to help individuals to understand and experience how much human welfare and nature conservation are interconnected (Rabinowitz et al., 2018; Wang et al., 2019). By promoting targeted and long‐term interventions (e.g., weekly mindfulness sessions, outdoor education) to governmental and nongovernmental institutions (UNESCO, OECD, school organisations, environmental education groups) and by training those who educate young people in these practices (teachers, educators, and parents), we believe that the desired outcomes (nature conservation and human well‐being) could be achieved at a moderate cost. As an example, “greening” schoolyards could improve people's HNC and well‐being and thereby foster support for sustainable policies, adding to its known positive effects on urban biodiversity and climate adaptation (the virtuous circle in Figure 1).

Surprisingly, we found little impact of environmental education on HNC, and we assume that this is due to the traditional anthropocentric, “rational” transmission of scientific knowledge, which has delegitimized and suppressed its emotional content (Buijs & Lawrence, 2013). Accordingly, and in line with previous studies (Tam, 2013), we found that HNC metrics that include multifaceted measures of HNC (i.e., both cognitive and affective) show the highest effect sizes. While this result remains to be further tested, studies in nonindustrialized populations (Atran et al., 2002) and children (Moore & Marcus, 2008) suggest that modifying the way scientific information is transmitted using nonanthropocentric knowledge about nonhuman species can have a positive impact on HNC. Examples of nonanthropocentric approaches, which would likely foster empathy and compassion toward the natural world (Mayer, 2018), include teaching children about the similarities between humans and other species (Clay & de Waal, 2013; Yokawa et al., 2019) and employing techniques to induce empathy toward all living beings (Berenguer, 2007).

If we are to develop effective research and conservation programmes that simultaneously support both people and nature worldwide, we urgently need longitudinal studies in children and in nonindustrial traditional cultures. Children from industrialized societies are known to show a strong affinity for nonhuman organisms (Moore & Marcus, 2008), but this affinity tends to fade with age (Hughes et al., 2019) and develop into low ecological concern at adulthood (Rosa et al., 2018) along with the acquisition of anthropocentric cultural norms (Wilks et al., 2020). We know much less about equivalent developmental changes in traditional societies, although proenvironmental behaviours may be more likely to persist into adulthood in societies with a high interdependency between humans and nature (Atran et al., 2002). Increased knowledge and appreciation of cultural norms and values and their development in diverse societies, both traditional and industrial, should contribute to better international environmental and educational policies and empower citizens and governments to achieve sustainable goals on a global scale.

## AUTHOR CONTRIBUTIONS

GBJ: conceptualization, methodology, analysis, writing‐original draft preparation, writing‐reviewing, and editing. CM: conceptualization, writing‐reviewing, and editing. CP: writing‐reviewing and editing, funding acquisition. MS: writing‐reviewing and editing. ML: conceptualization, writing‐reviewing, and editing, funding acquisition.

## ETHICS STATEMENT

This study complies with ethical scientific standards.

## CONFLICT OF INTEREST

The authors declare no conflict of interest.

## Supporting information

Supplementary information

## Data Availability

Data and code are freely available in the OSF repository at https://osf.io/ytrcn/?view_only=636530a6baab48cf9b8ac4f5daa204aa.
