# Supplementary material for: Human–nature connectedness as a pathway to sustainability: A global meta‐analysis
Source: Conserv Lett. 2021 Nov 21;15(1):e12852. doi: 10.1111/conl.12852 (PMC9286792; doi:10.1111/conl.12852)
Supplement: Supplementary file 1 — Supplementary information [file CONL-15-0-s001.docx]

**Supplementary information**

Appendix 1. Definitions of HNC

Leopold, a philosopher and ecologist (Leopold 1949), gave the first a representative definition of HNC when saying “we abuse land because we regard it as a commodity belonging to us. When we see land as a community to which we belong, we may begin to use it with love and respect.” For Leopold, it is necessary to understand a) “how humans see themselves as equal members of a large natural community”, b) “feel a sense of kinship with it”, c) “consider themselves as belonging to the natural world as much as it belongs to them”, d) and “consider their welfare as related to the well-being of the natural world”.

More recent definitions built upon Leopold’s definition and theoretical framework, have been proposed. Opotow (1996) defined HNC as “the degree of similarity we perceive between ourselves and other components of the natural world, and whether we consider nature and non-human natural entities to have standing as valued components of our social and moral community”. Schultz (Schultz 2002) who introduced the term connectedness with nature defined HNC as “the extent to which an individual includes nature within [their] cognitive representation of self”.

Finally, Mayer and Frantz (2004) recently defined HNC as “individual’s affective, experiential connection to nature” including “sense of kinship with plants and animals, sense of oneness with the natural world, and sense of equality between nature and the self”.

Several authors tried to describe the different facets of HNC. Schultz (2001) describes a *cognitive* component which refers to the extent to which humans integrated themselves in nature; an *affective* component which refers to ones’ sense of care for nature and a *behavioural* component which refer to one’s commitment to protect nature. Similarly, Ives et al. (2017) proposed five HNC categories including the *philosophical* connection (i.e. world view of what is nature and why it matters), the *emotional* connection (i.e. attachment or empathy for nature), the *cognitive* connection (i.e. knowledge or awareness of the environment), the *experiential* connection (i.e. contact with nature) and the *material* connection (i.e. consumption of goods/materials from nature).

However, those different categories changed from one study to another. Indeed, these categories are somehow subjective and arbitrary since one’s could pull some of them together (e.g., cognitive and philosophical both refers to knowledge individuals have about nature). In addition, items in the HNC measures (see below) often tackle more than one component of HNC and those measures also correlate with each other (Mayer & Frantz 2004) suggesting that they tackle the same underlying broader construct (Brügger et al. 2011; Tam 2013a; Capaldi et al. 2014). As such, we define HNC in its broader sense as *the extent to which humans include the natural world in the self or humans see themselves as part of nature.*

Appendix 2. Measures of HNC

Dunlap and Jones (2002) estimated that at least several hundred measures of human-nature connectedness have been developed since the 1960s across various research fields. Indeed, human nature connectedness has been used synonymously with nature connection, nature relatedness, love and care for nature, inclusion of nature in the self, connectedness to nature, connectivity with nature, empathy for nature, environmental identity, environmental concern, etc.

Among numerous measures, five major scales have been used more widely than others (see Appendix 1 for detailed items of the questionnaires). The **New Ecological Paradigm** (Dunlap et al. 2000), first labelled as New Environmental Paradigm (NEP), is the oldest and the most widely measure of environmental concern in the world. The NEP scale consists of 15 items and shows strong psychometrics and theoretical backgrounds. The original version of the NEP focuses on three components of HNC including beliefs about humanity’s ability to upset the balance of nature, the existence of limits to growth for human societies, and human domination of nature. The revised version includes five components: the reality of limits to growth, anti-anthropocentrism, the fragility of nature’s balance, rejection of exceptionalism and the possibility of an eco-crisis. The NEP has been shown to be an effective measure of HNC (Perrin & Benassi 2009). A recent meta-analysis reviewing 69 studies from 36 countries (including 58,279 participants from 139 samples) shows that variations in population type and selection of specific items in the NEP scale (i.e. sub-scales) have a significant effect on NEP scores suggesting that using all items from the scale is particularly important for reliability (Hawcroft & Milfont 2010).

In 2001, Schultz developed the **Inclusion of Nature in Self** (**INS**), a single-item measure that tackles self-identification with nature. Although very simple and easy to administrate, this scale nonetheless presents limits in its predictive validity (Martin & Czellar 2016). However, the INS test-retest correlations nonetheless present very high reliabilities between measurements of individual differences in connectedness with nature (Schultz et al. 2004).

The **Environmental Identity** scale (EID; Clayton 2003) is composed 24 items and is known to have good psychometrics properties (Olivos et al. 2011). The EID takes into account multiple components of HNC including a) individual’s interactions with nature, b) self-identification (e.g. humans as part of nature), c) ideology measured by support for environmental education and a sustainable lifestyle, d) positive emotions though aesthetic appreciation (“I would rather live in a small room or house with a nice view than a bigger room or house with a view of other buildings”) and e) autobiographical component, based on memories of interacting with nature (e.g. childhood exposure).

Similarly, the **Connectedness to Nature Scale** (CNS; Mayer & Frantz 2004) is composed of 14 items, has good psychometric properties since it correlates with related variables (e.g., correlations with the NEP scale) but not with confounding factors such as verbal ability or social desirability (Mayer & Frantz, 2004).

The most recent “classical” scale is **Nature Relatedness** (NR; Nisbet et al. 2009) which is composed of 21 items measures people understanding of interconnectedness between humans and other living beings.

All five scales measure the philosophical and cognitive components while the NR and the EID also tackle the affective components.

In parallel, other research areas from social sciences (e.g., geography) develop their own measurement of HNC that they called place attachment, sense of place or place identity (Jorgensen & Stedman 2001). Place attachment measures multidimensional constructs including a) beliefs about the relationship between self and place; b) feelings toward the place; and c) the behavioural exclusivity of the place in relation to alternatives. However, contrary to classical scales described above, PA is always associated with a specific place (e.g., specific lake) implying repeatability issues. For instance, as extensively described in a recent review on HNC (Ives et al. 2017), methodology and statistical analysis that have been done in studies measuring PA are not as “rigorous” as studies measuring classical scales. Similarly, sub-scales (e.g. selected items from classical scales) or other adapted scales focusing on specific non-human animals (e.g. spider or dolphin) or event (e.g. earth quake) have also been used, but selection of sub-items as well as generalization from one species or event to one another can also induce reliability issues (Hawcroft & Milfont 2010).

Other scales have been developed recently. For instance, the Dispositional Empathy for Nature scale tackles the emotional aspects of HNC (Tam 2013b) but only focus on the emotional components which do not corresponds to our definition of HNC. Finally, different other connection-with-nature scales including Disposition to Connect with Nature (Brügger et al. 2011), Commitment to Environment (Davis et al. 2009), implicit connection with nature have been developed (e.g. Implicit Associative Task (IAT) have also been used.

Appendix 3. Detailed items from the 5 classical scales

**1. New Ecological Paradigm (NEP, Dunlap et al., 1978, 2000)**

Instructions: Listed below are statements about the relationship between humans and the environment. For each one, please indicate whether you STRONGLY AGREE, MILDLY AGREE, are UNSURE, MILDLY DISAGREE or STRONGLY DISAGREE with it.

1.1 New Ecological Paradigm (original version) : 12 verbal items*

1. We are approaching the limit of the number of people the earth can support.

2. The balance of nature is very delicate and easily upset.

3. Humans have the right to modify the natural environment to suit their needs.

4. Mankind was created to rule over the rest of nature.

5. When humans interfere with nature it often produces disastrous consequences.

6. Plants and animals exist primarily to be used by humans.

7. To maintain a healthy economy we will have to develop a ‘‘steady–state’’ economy where industrial growth is controlled.

8. Humans must live in harmony with nature in order to survive.

9. The earth is like a spaceship with only limited room and resources

10. Humans need not adapt to the natural environment because they can remake it to suit their needs.

11. There are limits to growth beyond which our industrialized society cannot expand.

12. Mankind is severely abusing the environment.

*Agreement with 3, 4, 6, and 10 indicate anti-NEP responses.

1.2 Revised NEP : New Environmental Paradigm (Dunlap et al., 2000) : 15 verbal items^°^

1. We are approaching the limit of the number of people the earth can support.

2. Humans have the right to modify the natural environment to suit their needs.

3. When humans interfere with nature, it often produces disastrous consequences.

4. Human ingenuity will insure that we do NOT make the earth unliveable.

5. Humans are severely abusing the environment.

6. The earth has plenty of natural resources if we just learn how to develop them.

7. Plants and animals have as much right as humans to exist.

8. The balance of nature is strong enough to cope with the impacts of modern industrialised nations.

9. Despite our special abilities humans are still are still subject to the laws of nature.

10. The so-called “ecological crisis” facing humankind has been greatly exaggerated.

11. The Earth is like a spaceship with very limited room and resources

12. Humans were meant to rule over the rest of nature.

13. The balance of nature is very delicate and easily upset

14. Humans will eventually learn enough about how nature works to be able to control it.

15. If things continue on their present course, we will soon experience a major ecological catastrophe.

°Agreement with the eight odd-numbered items and disagreement with the seven even-numbered items indicate pro-nature responses.

**2. Inclusion of Nature in Self (INS ; adapted from Schultz, 2001) : 1 non-verbal item**

Instructions : Please circle the picture below which best describes your relationship with the natural environment. How interconnected are you with nature ?

**3. Environmental Identity (EID ;Clayton, 2003) : 24 verbal items**

Instructions : Please indicate the extent to which each of the following statements describes you by using the appropriate number from the scale below.

1 2 3 4 5 6 7 with 1 : Not at all true of me 4 : neither true nor untrue 7 : completely true of me

_____ 1. I spend a lot of time in natural settings (woods, mountains, desert, lakes, ocean).

_____ 2. Engaging in environmental behaviors is important to me.

_____ 3. I think of myself as a part of nature, not separate from it.

_____ 4. If I had enough time or money, I would certainly devote some of it to working for environmental causes.

_____ 5. When I am upset or stressed, I can feel better by spending some time outdoors "communing with nature".

_____ 6. Living near wildlife is important to me; I would not want to live in a city all the time.

_____ 7. I have a lot in common with environmentalists as a group.

_____ 8. I believe that some of today’s social problems could be cured by returning to a more rural lifestyle in which people live in harmony with the land.

_____ 9. I feel that I have a lot in common with other species.

_____ 10. I like to garden.

_____ 11. Being a part of the ecosystem is an important part of who I am.

_____ 12. I feel that I have roots to a particular geographical location that had a significant impact on my development.

_____ 13. Behaving responsibly toward the earth – living a sustainable lifestyle – is part of my moral code.

_____ 14. Learning about the natural world should be an important part of every child's upbringing.

_____ 15. In general, being part of the natural world is an important part of my selfimage.

_____ 16. I would rather live in a small room or house with a nice view than a bigger room or house with a view of other buildings.

_____ 17. I really enjoy camping and hiking outdoors.

_____ 18. Sometimes I feel like parts of nature – certain trees, or storms, or mountains – have a personality of their own.

_____ 19. I would feel that an important part of my life was missing if I was not able to get out and enjoy nature from time to time.

_____ 20. I take pride in the fact that I could survive outdoors on my own for a few days.

_____ 21. I have never seen a work of art that is as beautiful as a work of nature, like a sunset or a mountain range.

_____ 22. My own interests usually seem to coincide with the position advocated by environmentalists.

_____ 23. I feel that I receive spiritual sustenance from experiences with nature.

_____ 24. I keep mementos from the outdoors in my room, like shells or rocks or feathers.

**4A. Connectedness to Nature Scale (CNS ; Mayer & Frantz, 2004)**

Instructions: Please answer each of these questions in terms of the way you generally feel. There are no right or wrong answers. Using the following scale, in the space provided next to each question simply state as honestly and candidly as you can what you are presently experiencing.

1 2 3 4 5 with 1: Strongly disagree 3: Neutral 5: Strongly agree

____1. I often feel a sense of oneness with the natural world around me.

____2. I think of the natural world as a community to which I belong.

____3. I recognize and appreciate the intelligence of other living organisms.

____4. I often feel disconnected from nature.

____5. When I think of my life, I imagine myself to be part of a larger cyclical process of living.

____6. I often feel a kinship with animals and plants.

____7. I feel as though I belong to the Earth as equally as it belongs to me.

____8. I have a deep understanding of how my actions affect the natural world.

____9. I often feel part of the web of life.

____10. I feel that all inhabitants of Earth, human, and nonhuman, share a common ‘life force’.

____11. Like a tree can be part of a forest, I feel embedded within the broader natural world.

____12. When I think of my place on Earth, I consider myself to be a top member of a hierarchy that exists in nature.

____13. I often feel like I am only a small part of the natural world around me, and that I am no more important than the grass on the ground or the birds in the trees.

____14. My personal welfare is independent of the welfare of the natural world.

**4B. Adapted CNS for children: Connectedness to Nature Index (CNI ; Cheng & Monroe, 2012)**

1. I like to hear different sounds in nature

2. I like to see wild flowers in nature

3. When I feel sad, I like to go outside and enjoy nature

4. Being in the natural environment makes me feel peaceful

5. I like to garden

6. Collecting rocks and shells is fun

7. Being outdoors makes me happy

8. I feel sad when wild animals are hurt

9. I like to see wild animals living in a clean environment

10. I enjoy touching animals and plants

11. Taking care of animals is important to me

12. Humans are part of the natural world

13. People cannot live without plants and animals

14. Being outdoors makes me happy

15. My actions will make the natural world different

16. Picking up trash on the ground can help the environment

17. People do not have the right to change the natural environment

**5. Nature relatedness scale (NR; Nisbet et al., 2009): 21 verbal items**

Instructions: For each of the following, please rate the extent to which you agree with each statement, using the scale from 1 to 5 as shown below. Please respond as you really feel, rather than how you think “most people” feel.

1 Disagree strongly 2 Disagree a little 3 Neither Agree or disagree 4 Agree a little 5 Agree strongly

1. I enjoy being outdoors, even in unpleasant weather. ____

2. Some species are just meant to die out or become extinct. ____

3. Humans have the right to use natural resources any way we want. ____

4. My ideal vacation spot would be a remote, wilderness area. ____

5. I always think about how my actions affect the environment. ____

6. I enjoy digging in the earth and getting dirt on my hands. ____

7. My connection to nature and the environment is a part of my spirituality. ____

8. I am very aware of environmental issues. ____

9. I take notice of wildlife wherever I am. ____

10. I don’t often go out in nature. ____

11. Nothing I do will change problems in other places on the planet. ____

12. I am not separate from nature, but a part of nature. ____

13. The thought of being deep in the woods, away from civilization, is frightening. ____ 14. My feelings about nature do not affect how I live my life. ____

15. Animals, birds and plants should have fewer rights than humans. ____

16. Even in the middle of the city, I notice nature around me. ____

17. My relationship to nature is an important part of who I am. ____

18. Conservation is unnecessary because nature is strong enough to recover from any human impact. ____

19. The state of non-human species is an indicator of the future for humans. ____

20. I think a lot about the suffering of animals. ____

21. I feel very connected to all living things and the earth. ____

Scoring Information Reverse scored items: 2, 3, 10, 11, 13, 14, 15, 18; NR-self items: 5, 7, 8, 12, 14, 16, 17, 21; NR-perspective items: 2, 3, 11, 15, 18, 19, 20; NR-experience items: 1, 4, 6, 9, 10, 13 Overall NR score is calculated by averaging all 21 items (after reverse scoring appropriate items). Scores on the 3 NR dimensions are also calculated by averaging appropriate items after reverse scoring. A short-form version of the NR scale (NR-6) consists of items: 4, 5, 7, 9, 17, 21. Items are averaged to create a score on the brief measure of NR. No items are reverse scored.

Appendix 4. Additional method

1. Data search and sources

A total of 1697 citations were found using database searching: Citations were import from PubMed using R software while citations from Web of Science were import through their web platform (see Table S1 for keywords details). Additional 401 citations found in references of previous meta-analyses and reviews were also added. Finally, we pulled 2098 citations together in a single excel file (Figure S1).

2. Eligibility criteria

After removing manually duplicates (387 over 2098 records), we performed a three-stage screening to assess the relevance of the paper for future meta-analyses. In the first screening phase, titles and abstracts of the remaining 1711 records were screened and only *empirical, peer-reviewed* and *English-written* studies measuring *at least one HNC* were made eligible for inclusion. We thus excluded 931 irrelevant papers (i.e., off-topic), 27 non-peer-reviewed and conference papers, 240 reviews, opinion or perspective papers, 6 meta-analyses, and 26 papers not written in English (Figure S1).

In a second stage, we screened abstract and full text when necessary, to include only papers measuring *at least one factor* (causes and/or consequences) in addition to HNC measure. As such, papers aiming at developing or validating HNC scales (without factors) or a new version of a scale were excluded (n=29). We thus obtained 452 empirical papers including 5 case studies, 98 qualitative studies, 316 quantitative studies and 33 studies with both quantitative and qualitative data.

Since only *quantitative studies* can be further used in meta-analyses, full texts of those 348 quantitative studies were assessed for the final stage before inclusion. Among the 348 papers, 60 records reported more than one study or more than one HNC classical scale *shown to be repeatable namely NEP, INS, EID, CNS and NR*. After contacting corresponding authors to obtain missing data, we obtained 198 studies from 124 papers done by 69,763 participants.

3. Description of factors’ measurements for correlational data

We calculated R estimates between HNC and nine factors which corresponds to 4 broader categories described in the main text: (1) pro-environmental behaviours (self-reported behaviours and pro-environmental intentions), (2) pro-environmental values (biospheric, altruistic and egoistic values(Schultz 2001), utilitarian value of nature, interest in nature, ecological and environmental values), (3) Human health (mental and physical health and well-being, reversed effect sizes were used for negative mental health (e.g. depression) , negative personality trait (e.g. neuroticism) and negative physical health (e.g. physical stress), (4) Humanist values (social values, community values, moral values and humanist values), (5) materialistic values, 6) conservative (political) values), (7) environmental education (naturalist and scientific knowledge about nature), (8) exposure to real nature (time spent in nature, living close to nature, frequency of park visitation), and, (9) mindfulness (practicing mindfulness).

4. Description of factors’ measurements for experimental data

We calculated R estimates between HNC and 6 factors: 1) environmental education (naturalist and scientific knowledge about nature), 2) exposure to real nature (outdoor natural activities, walk in the forest, walk in indoor garden), 3) participation in an environmental program involving both outdoor activities and environmental knowledge 4) virtual nature (exposure to videos or pictures of nature), 5) Mindfulness and 6) a combination of mindfulness and real nature.

Since demographic information was often considered as a covariate to control, we did not include them as factor but performed appropriate statistics to investigate their impact on effect sizes (see SI Appendix 5 and Methods).

Appendix 5. Additional results

We investigated the effect of type of programs while controlling for gender ratio, experimental design, time of the post-test (immediately after intervention vs. more than two weeks later), duration of the program (≤1 day vs. ≥ 2 days) and age group on overall effect sizes of **experimental data**. To do so, we built a multivariate model with the five factors as fixed effects and lab and study and estimate ID as random effects (random = ~ 1|lab/study/estimate.id). We found an effect of the type of programs, the gender ratio and the duration of the program on the estimates (omnibus tests: program type: QM(df = 5) = 22.96, P = 0.0003; experimental design (pre-post vs. control/treatment) : QM(df = 1) = 1.84, P = 0.17; duration of the program (1 day or less; more than 2 days and one month): QM(df = 1) = 6.62, P= 0.01; time of the post-test (short term vs. retention): QM(df = 1) = 0.37, P = 0.54; age (children vs. adults): QM(df = 1) = 3.29, P = 0.070; gender ratio: QM(df = 1) = 4.73, P = 0.03; heterogeneity of the model: QE(df = 83) = 213.48, P < .0001; overall model: QM(df = 10) = 42.84, P = 0.001; see Figure 2, Methods, SI Appendix 5, Figure S3 and S4). We also investigated the effect of HNC metrics on overall effect sizes of **experimental data** while controlling for lab and study and estimate ID as random effects (random = ~ 1|lab/study/estimate.id). We did not find an effect of HNC metrics (omnibus tests: program type: QM(df = 3) = 1.84, P = 0.61).

We then investigated the effect of categories, region, gender ration, age group and HNC metrics on overall effect sizes of **correlational data**. To do so, we built a multivariate model with the five factors as fixed effects and lab and study and estimate ID as random effects. We found a significant effect of the category (Figure 2) and of the HNC metrics on effect sizes (omnibus tests: categories: QM(df = 3) = 179.83, P < .0001; age: QM(df = 1) = 0.48, P= 0.49; gender: QM(df = 2) = 0.86, P = 0.65; region: QM(df = 4) = 0.54, P = 0.97; HNC metrics: QM(df = 4) = 36.7, P < .0001; heterogeneity of the model: QE(df = 919) = 12588.43, P < .0001; overall model: QM(df = 14) = 221.01, P < .0001 (see Methods, Figure 2 and Figure S4). We then performed Tukey post-hoc tests to compare differences between HNC metrics. Uncorrected and corrected p values are reported in Table S4.

Appendix 6. Forest plots for experimental studies

Appendix 6.A Real Nature + Environmental education


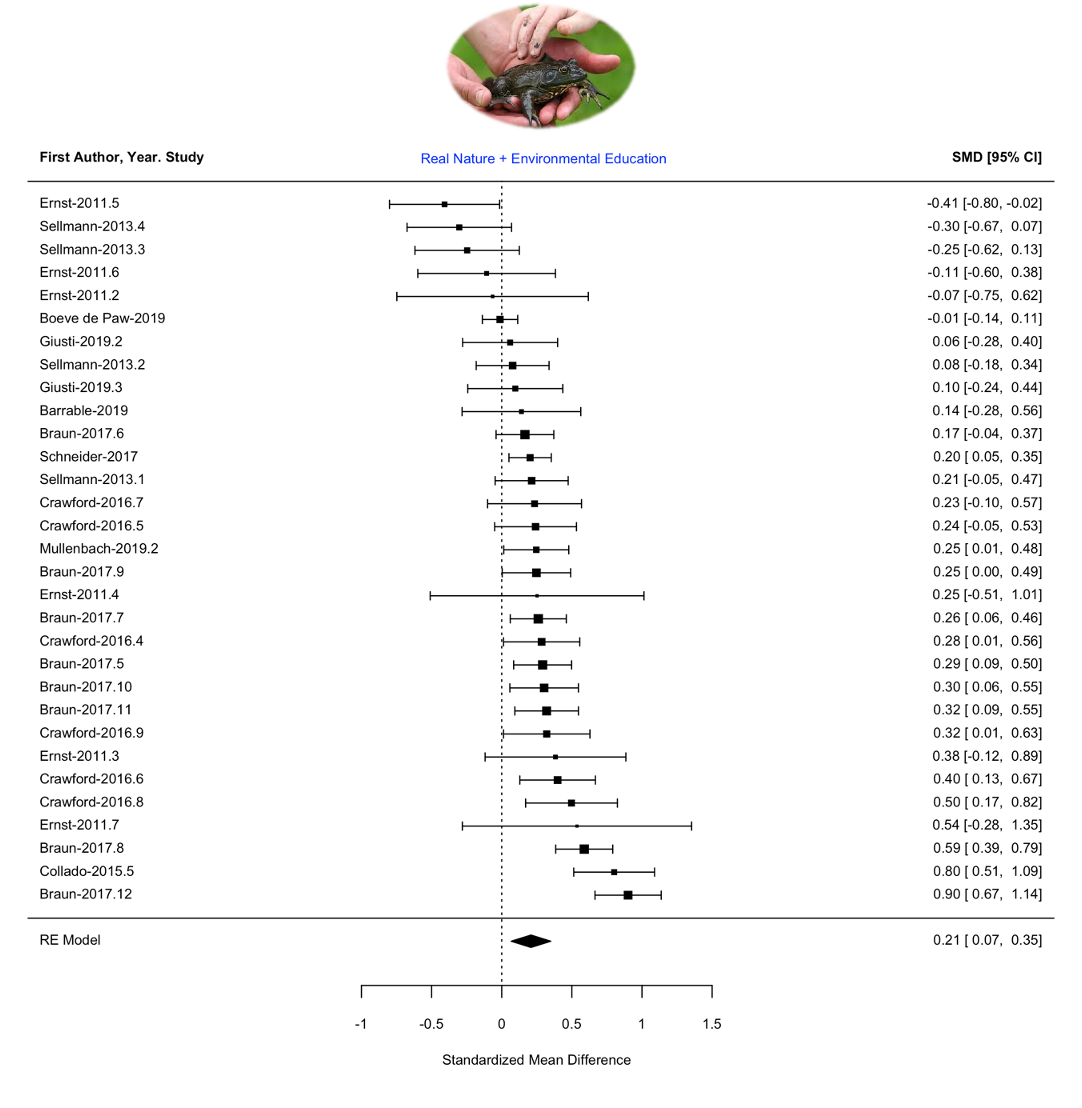


Appendix 6.B Real Nature

**
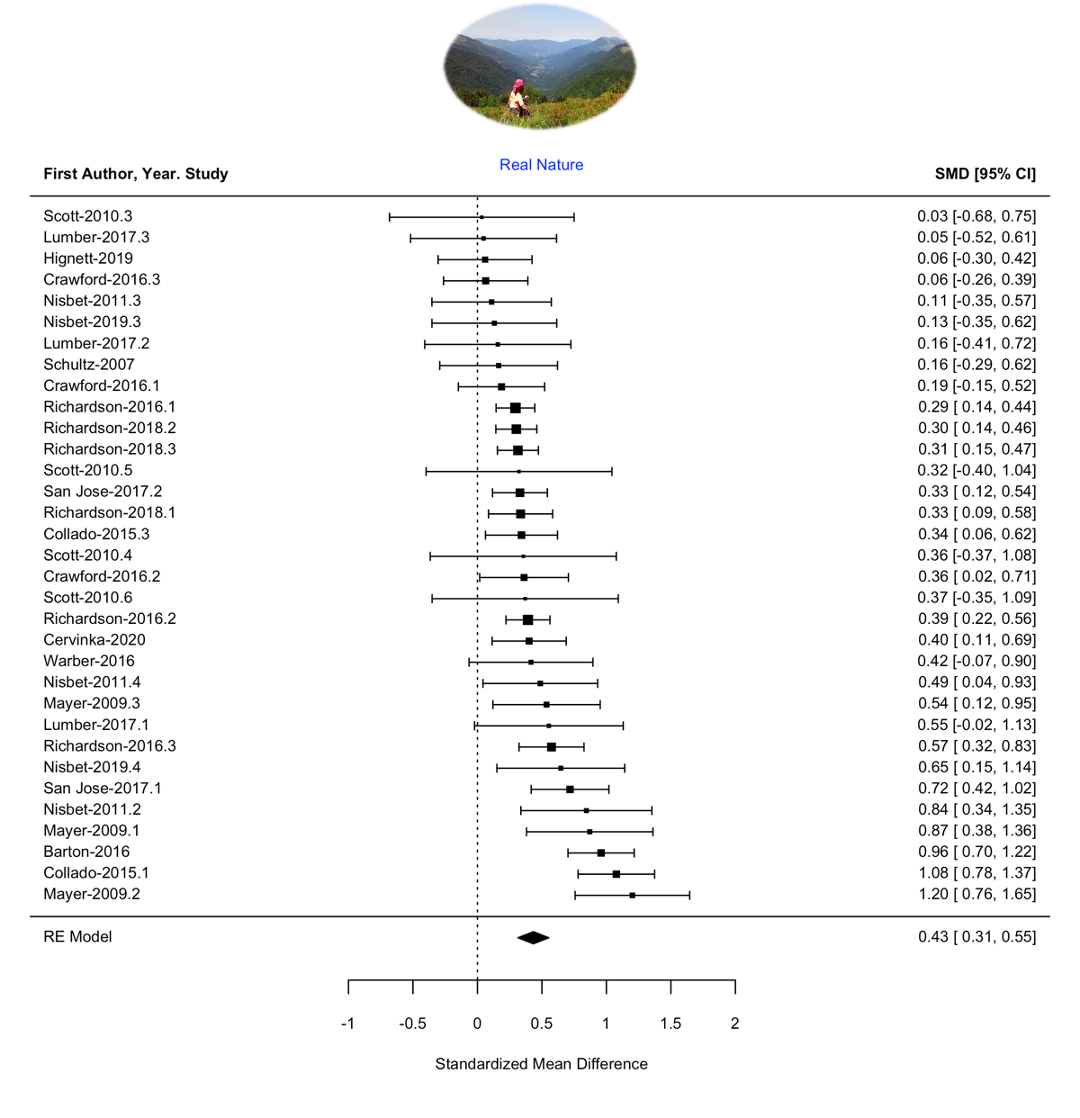
**

Appendix 6.C Real Nature + Mindfulness


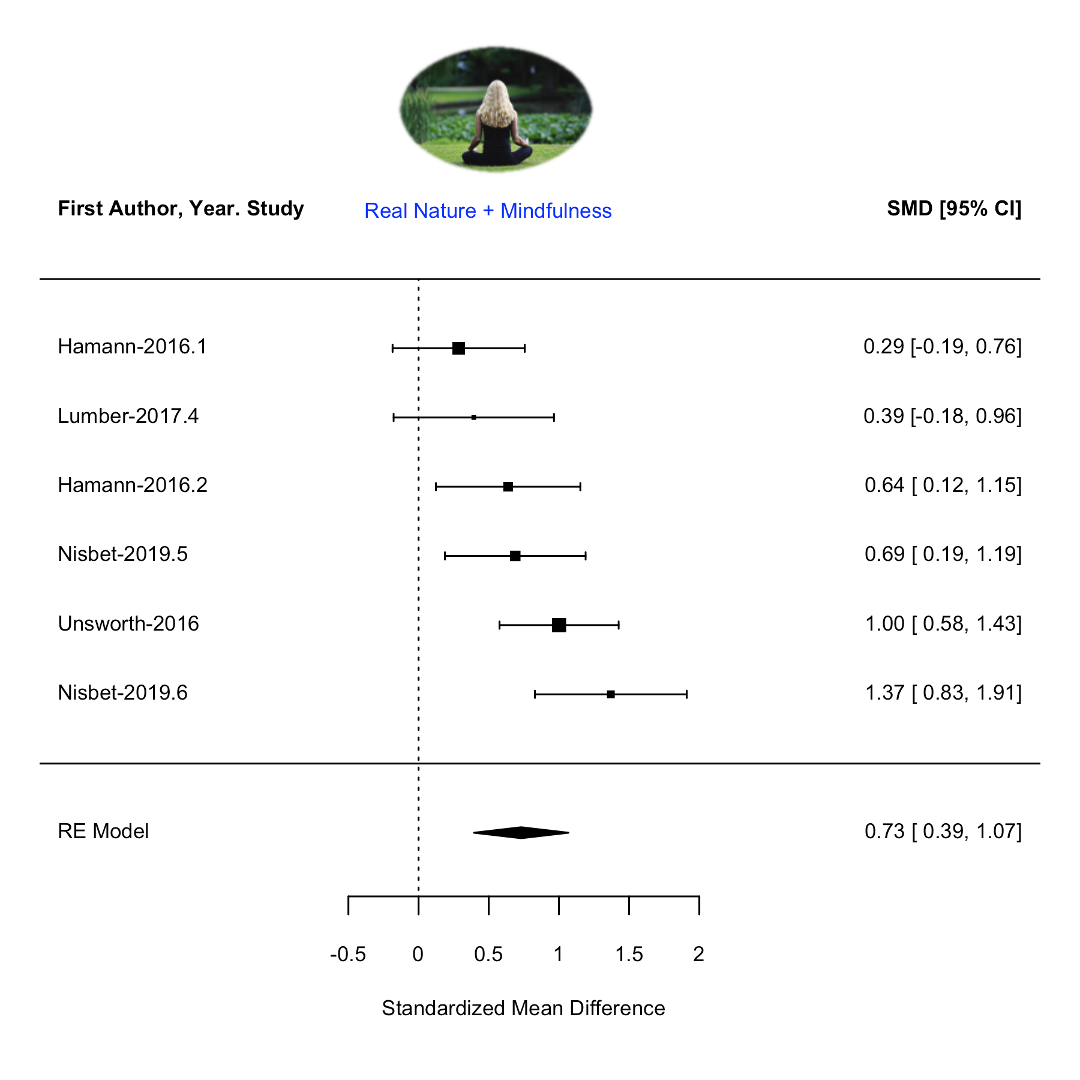


Appendix 6.D Environmental Education


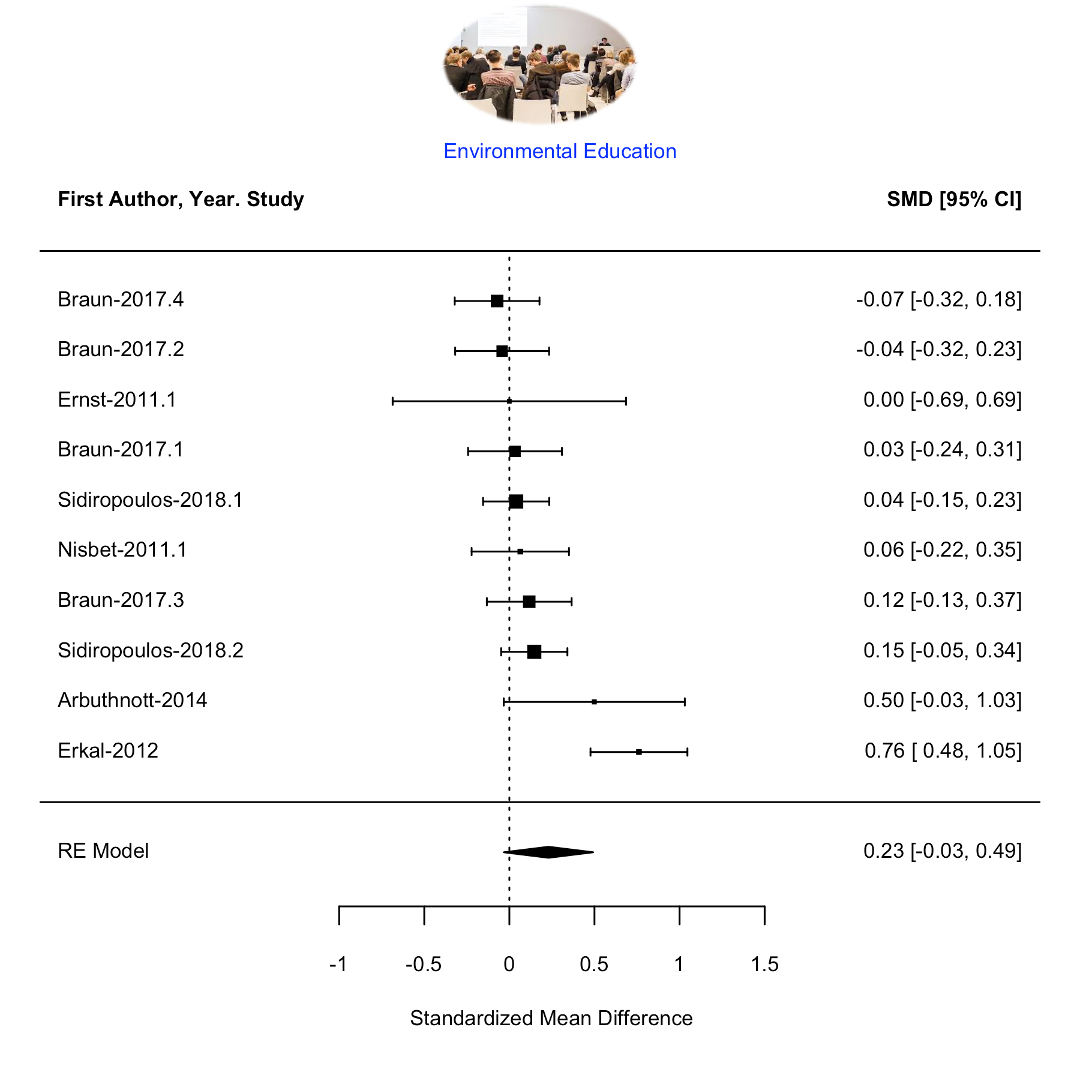


Appendix 6.E Virtual Nature


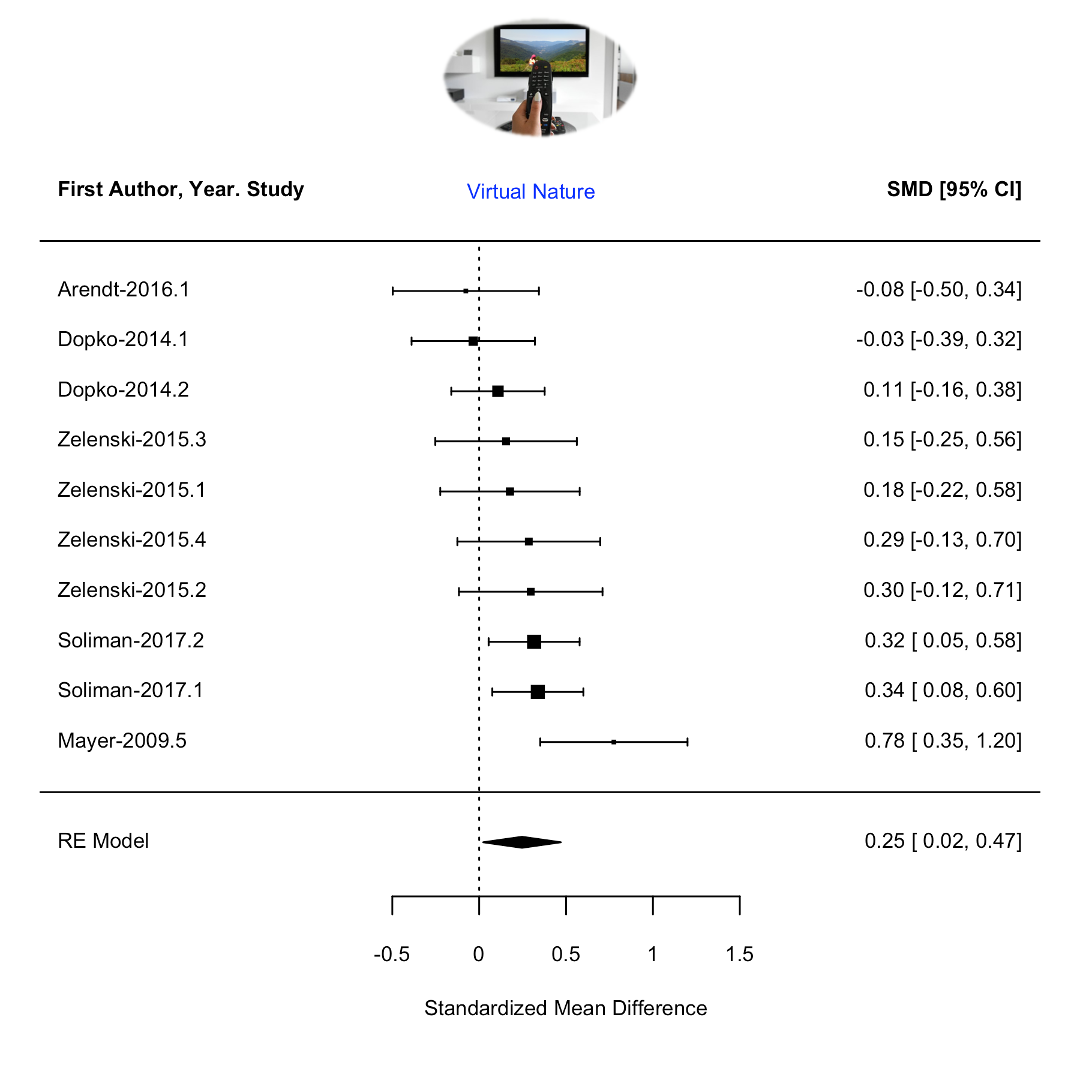


Appendix 6.F Mindfulness


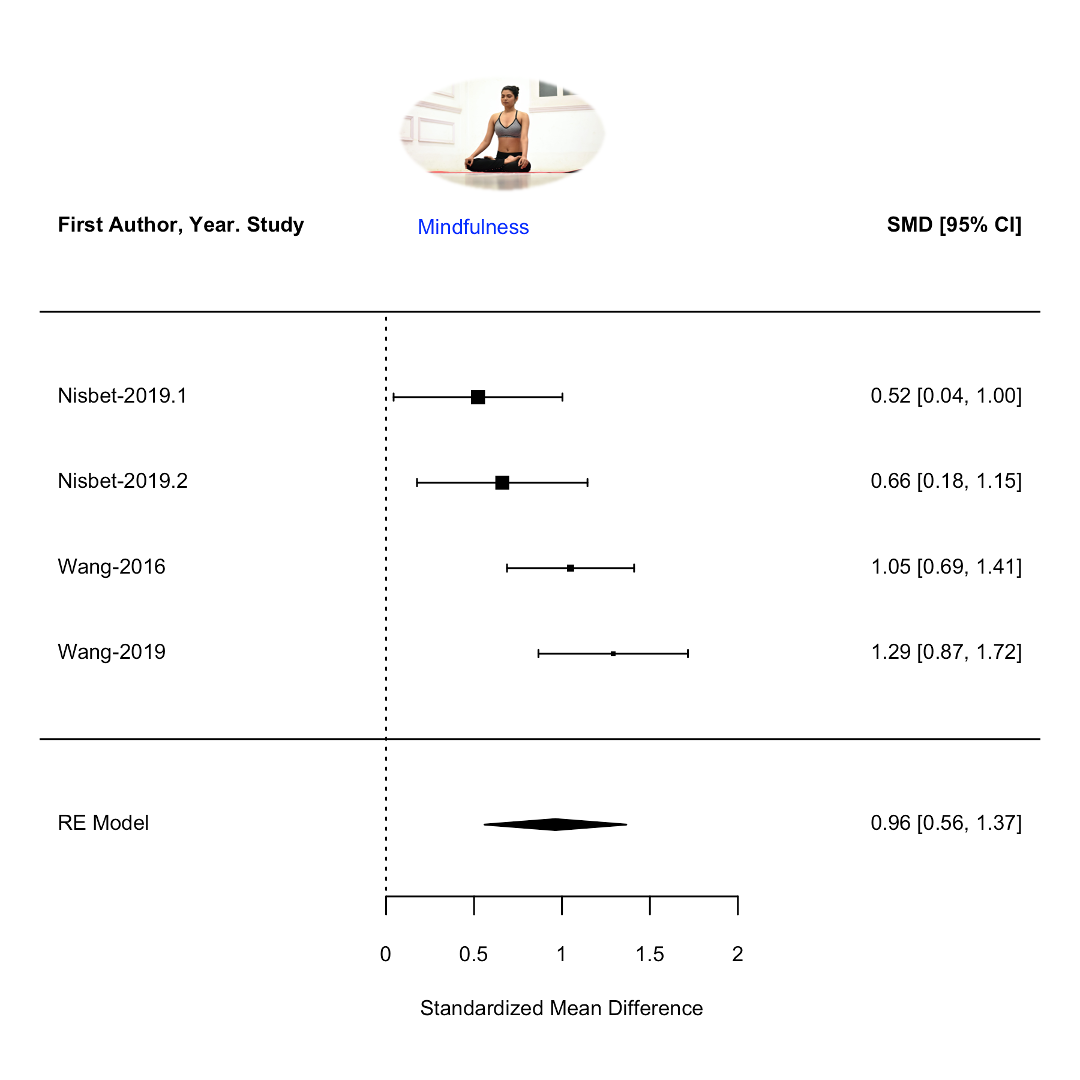


Table S1. Number of papers by keyword from database searching over the 1900-2020 (May) period

| **Keywords** | **Pubmed** | **Web of Science/MedLine** |
| --- | --- | --- |
| Human nature connectedness | 1 | 9 |
| Human nature connection | 3 | 17 |
| Empathy nature | 14 | 55 |
| Emotional affinity nature | 0 | 4 |
| Inclusion nature self | 0 | 4 |
| Environmental identity | 22 | 253 |
| Connectivity nature | 22 | 4 |
| Nature relatedness | 16 | 41 |
| Connect nature | 1 | 44 |
| Love of nature/Love and care for nature | 11 | 243 |
| Commitment to natural environment | 0 | 5 |
| Environmental connectedness | 33 | 33 |
| New Environmental Paradigm | 18 | 112 |
| Anthropomorphism nature | 0 | 6 |
| Anthropomorphism animal | 3 | 51 |
| Biophilia | 11 | 110 |
| Human plant relationship | 3 | 31 |
| Human-animal relationship | 75 | 390 |
| Human non-human relationship | 6 | 46 |

Table S2. Summary of meta-analyses from experimental data showing the R estimates, lower confidence interval at 95% (lCI), upper confidence intervals and 95% (uCI), number of papers n, number of effect sizes k, number of participants s, z and p values from the z-tests for each category as well as Q (residual heterogeneity) and its corresponding p value Qp for each model (one model per line) with lab and study as random effects.

| **Type of programs** | **R** | **lCI** | **uCI** | **n** | **k** | **s** | **z** | **p** | **Q** | **Qp** |
| --- | --- | --- | --- | --- | --- | --- | --- | --- | --- | --- |
| **Contact with real nature** | 0.18 | 0.13 | 0.23 | 25 | 70 | 4773 | 6.5 | <.00001 | 246 | <.00001 |
| *real nature + environmental education* | 0.1 | 0.034 | 0.17 | 10 | 31 | 2352 | 2.9 | 0.004 | 109 | <.00001 |
| *real nature* | 0.21 | 0.15 | 0.27 | 15 | 33 | 2290 | 7.1 | <.00001 | 86 | <.00001 |
| *real nature + mindfulness* | 0.34 | 0.19 | 0.48 | 4 | 6 | 220 | 4.3 | <.0001 | 12 | 0.037 |
| **Other programs (without real nature)** | 0.19 | 0.089 | 0.30 | 14 | 24 | 2328 | 3.5 | <.001 | 94 | <.00001 |
| *environmental education* | 0.11 | -0.016 | 0.24 | 6 | 10 | 909 | 1.7 | 0.085 | 26 | 0.0017 |
| *virtual nature* | 0.12 | 0.012 | 0.23 | 5 | 10 | 1113 | 2.2 | 0.03 | 13 | 0.17 |
| *mindfulness* | 0.43 | 0.26 | 0.58 | 3 | 4 | 306 | 4.7 | <.0001 | 20 | <.00001 |

*Note. Real nature + Environmental Education: Outdoor activities including scientific knowledge. Real Nature: Outdoor programs or experimental manipulations using exposure to real nature; Virtual nature: videos or pictures of nature administrate in an indoor setting. EE: educational programs or experimental manipulations involving transmission of naturalist or ecological knowledge in an indoor setting. Mindfulness: focusing on one self or surrounds in the present moment in an indoor setting; Real Nature + Mindfulness: practicing mindfulness in real nature.*

Table S3. Summary of meta-analyses from correlational data showing the R estimates, lower confidence interval at 95% (lCI), upper confidence intervals and 95% (uCI), number of papers n, number of effect sizes k, number of participants s, z and p values from the z-tests for each category as well as Q (residual heterogeneity) and its corresponding p value Qp for each model (one model per line) with lab and study as random effects.

| **Category** | **R** | **lCI** | **uCI** | **n** | **k** | **s** | **z** | **p** | **Q** | **Qp** |
| --- | --- | --- | --- | --- | --- | --- | --- | --- | --- | --- |
| **Nature conservation** | 0.36 | 0.32 | 0.39 | 84 | 404 | 51633 | 18 | <.00001 | 9713 | <.00001 |
| *pro-environmental behavior* | 0.35 | 0.32 | 0.39 | 54 | 168 | 36945 | 17 | <.00001 | 2098 | <.00001 |
| *pro-environmental values* | 0.36 | 0.31 | 0.41 | 52 | 231 | 33793 | 12 | <.00001 | 8434 | <.00001 |
| **Human welfare** | 0.23 | 0.20 | 0.27 | 50 | 477 | 30343 | 12 | <.00001 | 3422 | <.00001 |
| *Human health* | 0.22 | 0.19 | 0.25 | 47 | 430 | 28558 | 13 | <.00001 | 2587 | <.00001 |
| *Humanist and social values* | 0.28 | 0.10 | 0.45 | 8 | 38 | 3762 | 3 | 0.003 | 751 | <.00001 |
| **Lifestyle characteristics** | 0.27 | 0.21 | 0.32 | 35 | 93 | 14335 | 8.8 | <.00001 | 1508 | <.00001 |
| *Environmental education* | 0.25 | 0.14 | 0.35 | 10 | 11 | 6570 | 4.5 | <.00001 | 209 | <.00001 |
| *Real nature* | 0.26 | 0.18 | 0.33 | 25 | 68 | 10304 | 6.7 | <.00001 | 1149 | <.00001 |
| *Mindfulness* | 0.32 | 0.21 | 0.43 | 9 | 14 | 2539 | 5.3 | <.00001 | 89 | <.00001 |
| **Non-environmental values** | -0.23 | -0.29 | -0.17 | 7 | 12 | 3174 | -7.3 | <.00001 | 34 | <.0001 |
| *materialistic values* | -0.23 | -0.31 | -0.15 | 4 | 7 | 1324 | -5.5 | <.00001 | 11 | 0.082 |
| *Conservative political values* | -0.23 | -0.34 | -0.11 | 3 | 5 | 1850 | -3.8 | 0.0001 | 23 | 0.0001 |

*Note. Nature conservation: pro-environmental values (ecological, personal interest and appreciation of nature) and pro-environmental behaviours (self-reported and observed pro-environmental behaviours as well as pro-environmental intentions). Human welfare: psychological health (mental health, positive personality traits, academic performance), social well-being (happiness, social and psychological well-being), physical health (vitality, mobility) and humanist values (value of human beings including human freedom and progress, individually and collectively namely social values, community values, moral values and humanist values). Reversed effect sizes were used for negative mental health (e.g., depression), negative personality trait (e.g., neuroticism) and negative physical health (e.g., physical stress). lifestyle characteristics: contact with real nature (e.g., time spent in nature), environmental education (scientific or naturalist knowledge) and mindfulness practices (focusing on the present moment). non-environmental values: materialistic and utilitarian values (values focusing on wealth, possessions, image, and status including consumerism) and conservatism (political values emphasizing traditional social institutions).*

| **Experimental studies** | | | | | |
| --- | --- | --- | --- | --- | --- |
| *Overall* | CNS | NR | INS | NEP | EID |
| CNS | **-** | 0.93 | 0.41 | 0.63 | - |
| NR |  | - | 0.49 | 0.73 | - |
| INS |  |  | - | 0.29 | - |
| NEP |  |  |  | - | - |
| EID |  |  |  |  | - |
| **Correlational studies** | | | | | |
| *Overall* | CNS | NR | INS | NEP | EID |
| CNS | **-** | 0.11 | 0.062 | **0.003** | **0.0006*** |
| NR |  | - | **0.0006*** | **0.0002*** | **0.012** |
| INS |  |  | - | 0.39 | **<.0001*** |
| NEP |  |  |  | - | **<.0001*** |
| EID |  |  |  |  | - |
| *Nature conservation above the diagonal and human welfare below the diagonal* | CNS | NR | INS | NEP | EID |
| CNS | **-** | **<.001*** | 0.17 | 0.12 | **0.001*** |
| NR | 0.16 | - | **<.0001*** | **<.0001*** | 0.53 |
| INS | 0.18 | 0.88 | - | 0.82 | **<.001*** |
| NEP | **0.01** | 0.35 | 0.46 | - | **<.001*** |
| EID | 0.86 | 0.58 | 0.56 | 0.40 | - |
| *Lifestyle characteristics above the diagonal and non-environmental values below the diagonal* | CNS | NR | INS | NEP | EID |
| CNS | **-** | 0.30 | 0.20 | 0.23 | 0.13 |
| NR | - | - | **0.008** | 0.06 | 0.35 |
| INS | 0.38 | - | - | 0.79 | **0.017** |
| NEP | 0.99 | - | 0.43 | - | **0.034** |
| EID | 0.48 | - | 0.99 | 0.44 | - |

Table S4. Post-hoc comparisons (Tukey tests) of HNC metrics from experimental and correlational (overall and by meta-analysis) studies showing uncorrected p-values.

*Note. Uncorrected significant P values are bolded. *: significant p values after Bonferroni correction.*


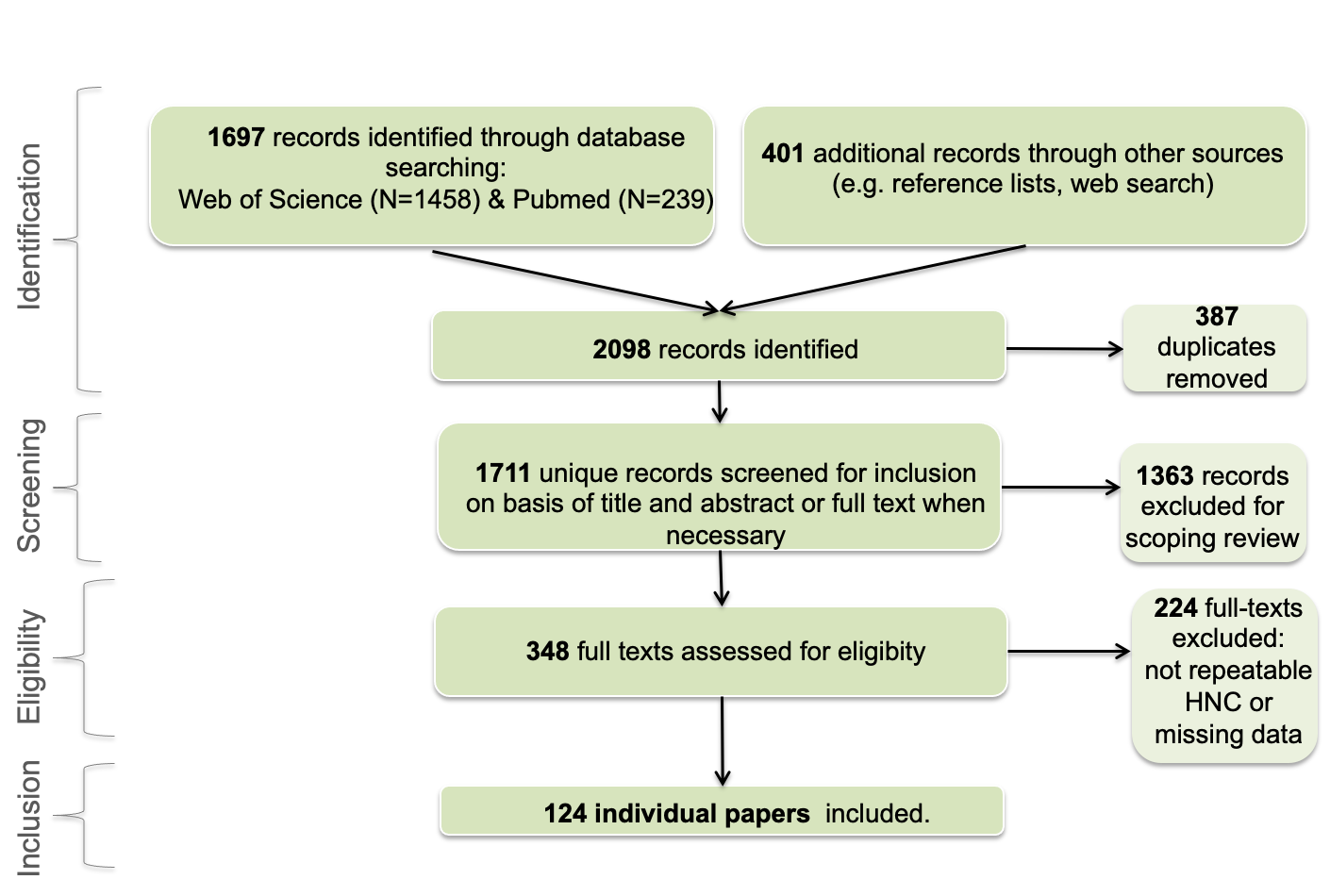
 Figure S1. PRISMA flow diagram.

Figure S2. Description of all studies included in the review. A) Number of participants for children (<18 years old), young adults (18 to 25 years old), adults (26 to 40) and older adults (>40). B) Number of participants in function of HNC metrics. C) Number of participants by region is presented on the left while coloured map representing the number of participants by country, with grey referring to country without any data on the right. The EID scale was the least used scale. Most of the studies are strongly biased towards adults from industrialized countries.

Figure S3. R estimates from experimental by duration of the treatment (less than one day vs. more than two days) and time of the post-test (immediately after intervention vs. more than 2 weeks after intervention). Each round represents the R estimate for each HNC metrics with n, k and s referring to the number of papers, number of effect sizes and number of participants respectively. Horizontal lines indicate the 95% confidence intervals for each factor and cross the vertical dot lines when non-significant.

**
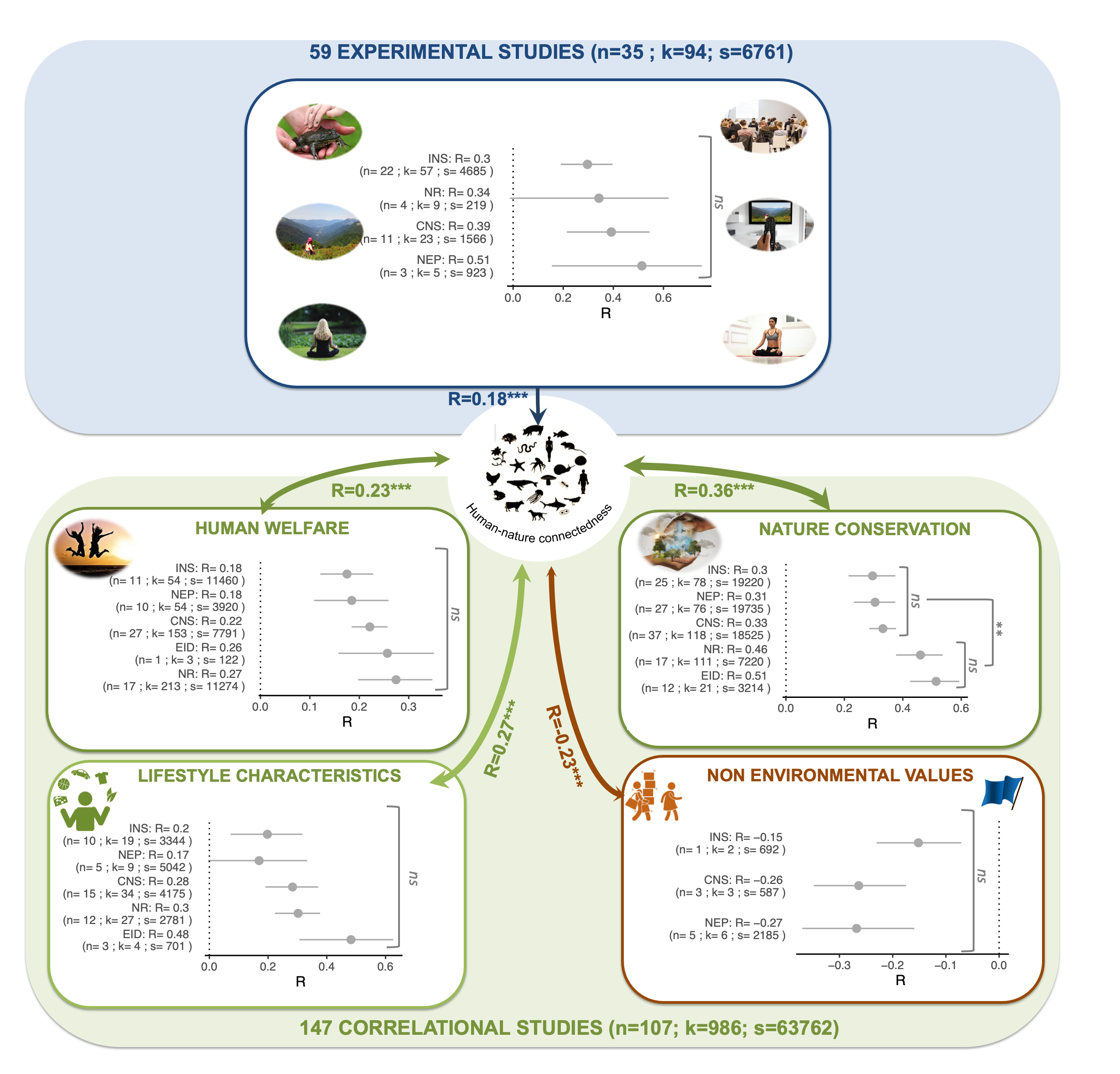
**

Figure S4. R estimates from experimental and correlational data by Human-Nature Connectedness (HNC) metrics. Each round represents the R estimate for each HNC metrics with n, k and s referring to the number of papers, number of effect sizes and number of participants respectively. Horizontal lines indicate the 95% confidence intervals for each factor and cross the vertical dot lines when non-significant. *** P<.0001; **P<.001; ns: non-significant. P values contrasting HNC metrics were corrected for multiple comparisons (see Appendix 5 and Table S4 for details).

**
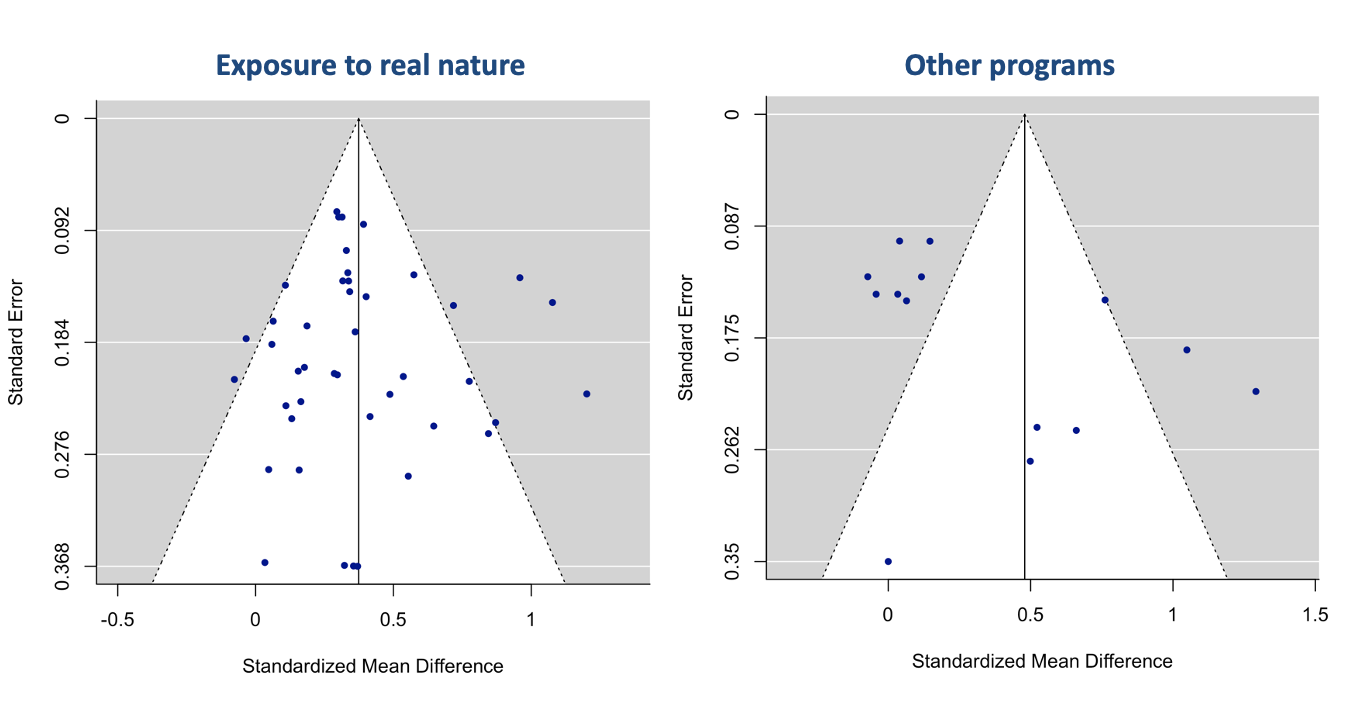
**

Figure S5. Funnels plots from the meta-analyses of experimental studies. Each dot represents effect size from one study.

**
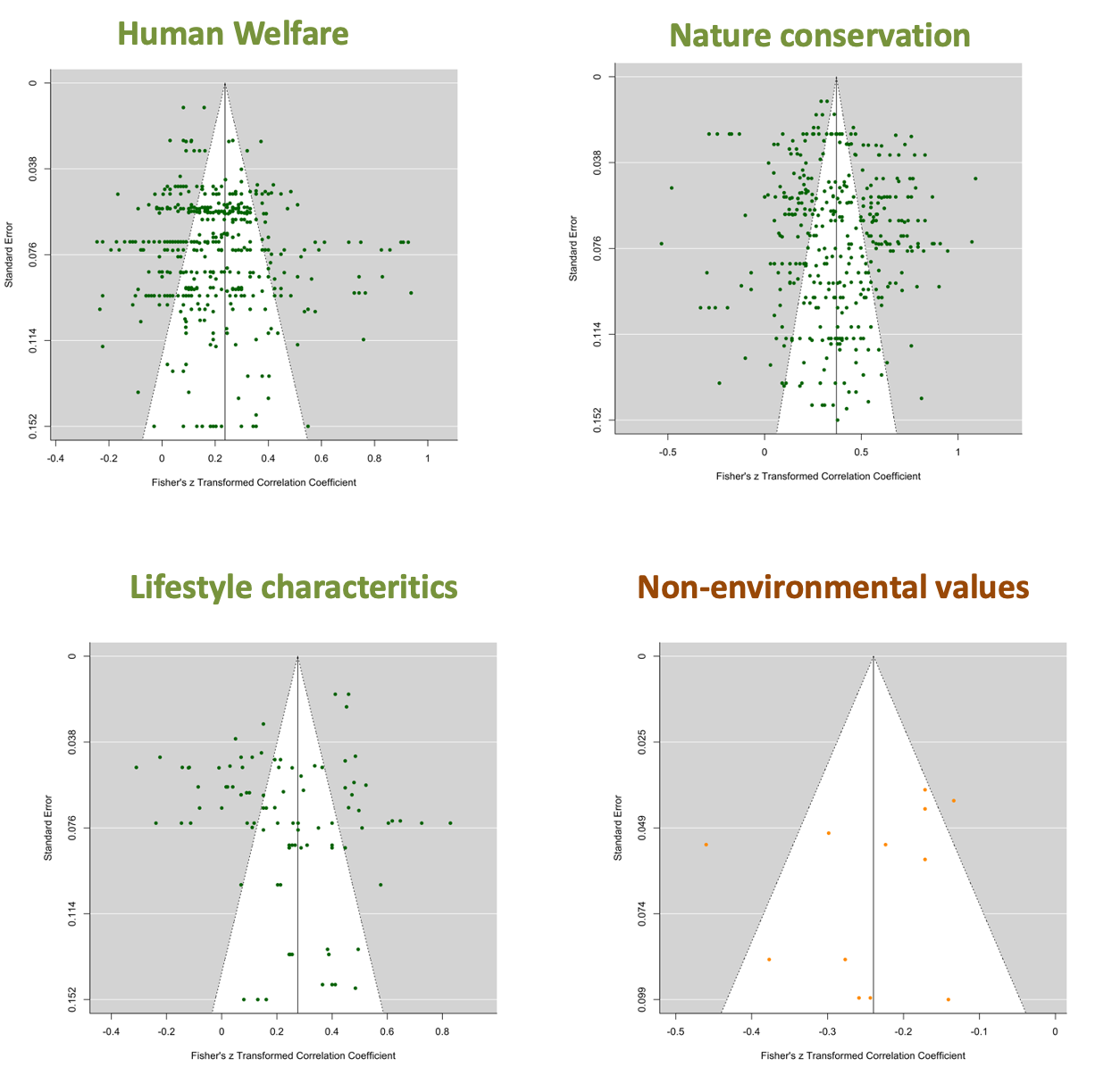
**

Figure S6. Funnels plots from the meta-analyses of correlational studies. Each dot represents effect size from one study.


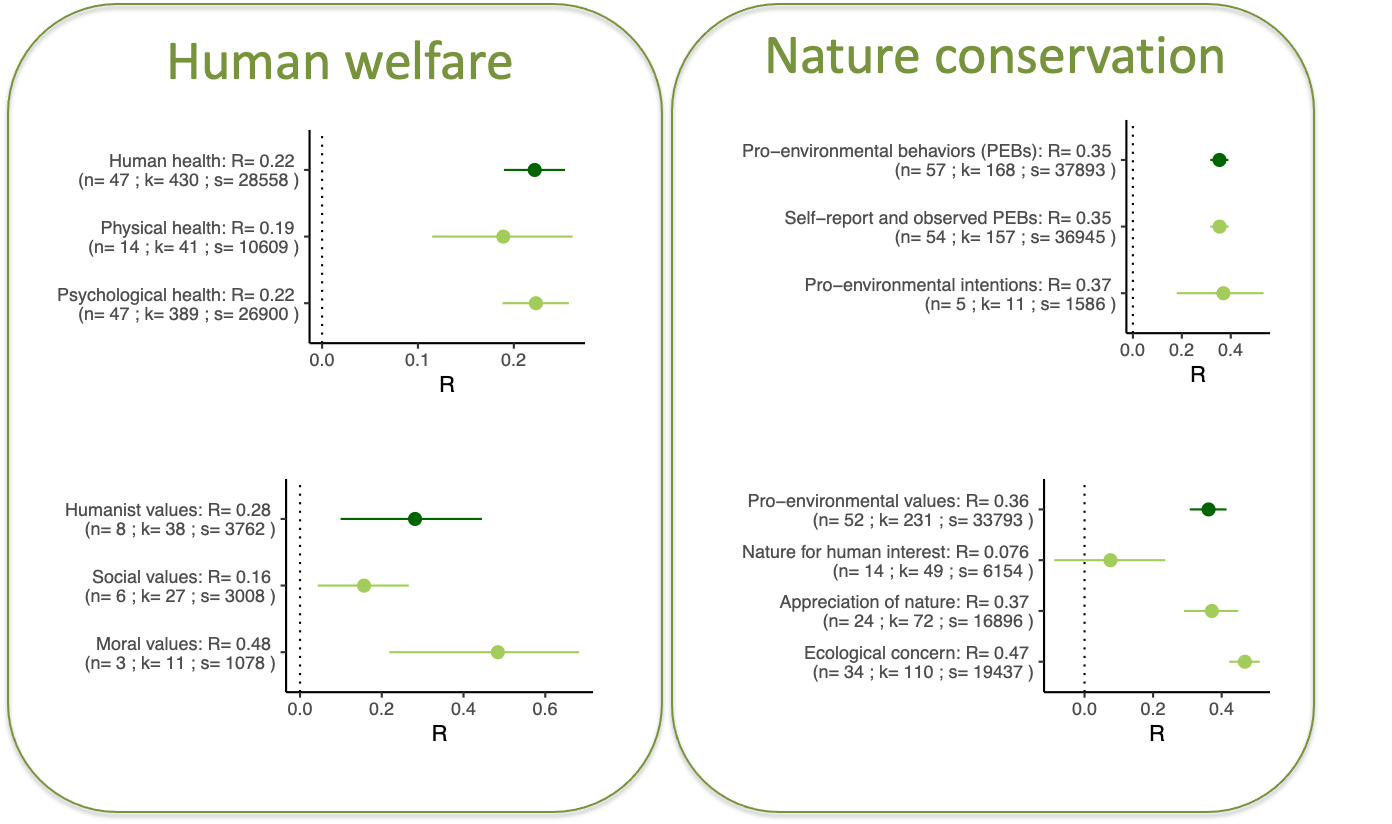


Figure S7. R estimates for each sub-category from human welfare (human health include physical and psychological health and humanist values include social and moral values) and nature conservation (pro-environmental behaviour (PEB) include observed and self-reported PEB and pro-environmental intentions while pro-environmental values include nature for human interest, appreciation of nature and ecological concern). Each round represents the R estimate for each factor with n, k and s referring to the number of papers, number of effect sizes and number of participants respectively. Horizontal lines indicate the 95% confidence intervals for each factor and cross the vertical dot lines when non-significant.

**Supplementary information’s references**

Brügger, A., Kaiser, F.G. & Roczen, N. (2011). Connectedness to Nature, Inclusion of Nature, Environmental Identity, and Implicit Association with Nature. *Eur. Psychol.*, 16, 324–333.

Capaldi, C.A., Dopko, R.L. & Zelenski, J.M. (2014). The relationship between nature connectedness and happiness: a meta-analysis. *Front. Psychol.*, 5.

Clayton, S. (2003). Environmental identity: A conceptual and an operational definition. *Identity Nat. Environ. Psychol. Significance Nat.*, 45–65.

Davis, J.L., Green, J.D. & Reed, A. (2009). Interdependence with the environment: Commitment, interconnectedness, and environmental behavior. *J. Environ. Psychol.*, 29, 173–180.

Dunlap, R. & Jones, R. (2002). Environmental concern: Conceptual and measurement issues. In Handbook of environmental sociology, ed. R. Dunlap and W. Michelson. London: Greenwood.

Dunlap, R.E., Liere, K.D.V., Mertig, A.G. & Jones, R.E. (2000). New Trends in Measuring Environmental Attitudes: Measuring Endorsement of the New Ecological Paradigm: A Revised NEP Scale. *J. Soc. Issues*, 56, 425–442.

Hawcroft, L.J. & Milfont, T.L. (2010). The use (and abuse) of the new environmental paradigm scale over the last 30 years: A meta-analysis. *J. Environ. Psychol.*, 30, 143–158.

Ives, C.D., Giusti, M., Fischer, J., Abson, D.J., Klaniecki, K., Dorninger, C., Laudan, J., Barthel, S., Abernethy, P., Martín-López, B., Raymond, C.M., Kendal, D. & von Wehrden, H. (2017). Human–nature connection: a multidisciplinary review. *Curr. Opin. Environ. Sustain.*, Open issue, part II, 26–27, 106–113.

Jorgensen, B.S. & Stedman, R.C. (2001). Sense of place as an attitude: Lakeshore owners attitudes toward their properties. *J. Environ. Psychol.*, 21, 233–248.

Leopold, A. (1949). *A Sand County almanac, and sketches here and there*. Oxford University Press, USA.

Martin, C. & Czellar, S. (2016). The extended inclusion of nature in self scale. *J. Environ. Psychol.*, 47, 181–194.

Mayer, F.S. & Frantz, C.M. (2004). The connectedness to nature scale: A measure of individuals’ feeling in community with nature. *J. Environ. Psychol.*, 24, 503–515.

Nisbet, E.K., Zelenski, J.M. & Murphy, S.A. (2009). The Nature Relatedness Scale: Linking Individuals’ Connection With Nature to Environmental Concern and Behavior. *Environ. Behav.*, 41, 715–740.

Olivos, P., Aragonés, J.I. & Amérigo, M. (2011). The connectedness to nature scale and its relationship with environmental beliefs and identity. *Int. J. Hisp. Psychol.*, 4, 5–19.

Opotow, S. (1996). Is Justice Finite? The Case of Environmental Inclusion. In: *Curr. Soc. Concerns Justice*, Critical Issues in Social Justice (eds. Montada, L. & Lerner, M.J.). Springer US, Boston, MA, pp. 213–230.

Perrin, J.L. & Benassi, V.A. (2009). The connectedness to nature scale: A measure of emotional connection to nature? *J. Environ. Psychol.*, 29, 434–440.

Schultz, P.W. (2001). The structure of environmental concern: Concern for self, other people, and the biosphere. *J. Environ. Psychol.*, 21, 327–339.

Schultz, P.W. (2002). Inclusion with nature: The psychology of human-nature relations. In: *Psychol. Sustain. Dev.* Springer, pp. 61–78.

Tam, K.-P. (2013a). Concepts and measures related to connection to nature: Similarities and differences. *J. Environ. Psychol.*, 34, 64–78.

Tam, K.-P. (2013b). Dispositional empathy with nature. *J. Environ. Psychol.*, 35, 92–104.
